# Factors Associated with Early Mortality in Critically Ill Patients Following the Initiation of Continuous Renal Replacement Therapy

**DOI:** 10.3390/jcm7100334

**Published:** 2018-10-08

**Authors:** Youn Kyung Kee, Dahye Kim, Seung-Jung Kim, Duk-Hee Kang, Kyu Bok Choi, Hyung Jung Oh, Dong-Ryeol Ryu

**Affiliations:** 1 Department of Internal Medicine, Hangang Sacred Heart Hospital, Hallym University College of Medicine, Seoul 07247, Korea; no7766@yuhs.ac; 2 Department of Nursing, Ewha Womans University Mokdong Hospital, Seoul 07985, Korea; dahye7738@gmail.com; 3 Department of Internal Medicine, School of Medicine, Ewha Womans University, Seoul 03761, Korea; sjkimwon@ewha.ac.kr (S.-J.K.); dhkang@ewha.ac.kr (D.-H.K.); kbchoi@ewha.ac.kr (K.B.C.); 4 Research Institute for Human Health Information, Ewha Womans University Mokdong Hospital, Seoul 07985, Korea; 5 Ewha Institute of Convergence Medicine, Ewha Womans University Mokdong Hospital, Seoul 07985, Korea; 6 Tissue Injury Defense Research Center, College of Medicine, Ewha Womans University, Seoul 07985, Korea

**Keywords:** continuous renal replacement therapy, early mortality, clinical illness

## Abstract

Continuous renal replacement therapy (CRRT) is an important modality to support critically ill patients, and the need for CRRT treatment has been increasing. However, CRRT management is costly, and the associated resources are limited. Thus, it remains challenging to identify patients that are likely to have a poor outcome, despite active treatment with CRRT. We sought to elucidate the factors associated with early mortality after CRRT initiation. We analyzed 240 patients who initiated CRRT at an academic medical center between September 2016 and January 2018. We compared baseline characteristics between patients who died within seven days of initiating CRRT (early mortality), and those that survived more than seven days beyond the initiation of CRRT. Of the patients assessed, 130 (54.2%) died within seven days of CRRT initiation. Multivariate logistic regression models revealed that low mean arterial pressure, low arterial pH, and high Sequential Organ Failure Assessment score before CRRT initiation were significantly associated with increased early mortality in patients requiring CRRT. In conclusion, the mortality within seven days following CRRT initiation was very high in this study. We identified several factors that are associated with early mortality in patients undergoing CRRT, which may be useful in predicting early outcomes, despite active treatment with CRRT.

## 1. Introduction

Acute kidney injury (AKI) is a major complication in critically ill patients, and it is associated with high mortality [1,2,3]. Continuous renal replacement therapy (CRRT) is a widely chosen treatment option in AKI patients requiring renal replacement therapy (RRT), particularly for hemodynamically unstable patients with considerable fluid accumulation [4,5,6]. Although CRRT is commonly considered as an initial option for critically ill patients who need RRT, the cost of CRRT treatment is higher than that of intermittent RRT, and CRRT-associated resources are limited [7,8].

Despite advances in CRRT techniques over the last several years, the mortality rate of patients undergoing CRRT remains high [9,10,11,12]. Many studies have investigated the benefit of CRRT treatment with regard to clinical outcomes and assessed potential prognostic factors for mortality [13,14,15]. Several factors, including sepsis, high acute physiology, and chronic health evaluation (APACHE) II score and/or sequential organ failure assessment (SOFA) score, poor urine output before CRRT initiation, comatose state, need for mechanical ventilation, fluid overload status, and type of CRRT solution are associated with increased mortality rate [16,17,18,19,20,21,22]. However, some patients die within one week of CRRT initiation, causing physicians to often doubt the benefits of such an invasive procedure on patient survival and/or renal preservation. Unfortunately, there are few studies to determine which factors are associated with increased early mortality in critically ill patients undergoing CRRT.

Considering the high cost and the limited resources available, identification of patients who would be more likely to have a poor outcome despite active treatment with CRRT is necessary to make an informed decision for patients requiring RRT. Thus, the aim of this study was to investigate the factors that are associated with increased early mortality, which we defined as death in the 7 days following CRRT initiation.

## 2. Methods

### 2.1. Study Population

This was a retrospective observational study of patients aged 18 years or older who initiated CRRT at a tertiary academic medical center between September 2016 and January 2018. Patients who were younger than 18 years, who were undergoing chronic dialysis due to end-stage renal disease, or who had a less than 3 month life expectancy due to malignancy were excluded. Ultimately, 240 patients were enrolled and assessed to determine the factors that were associated with early mortality in critically ill patients undergoing CRRT. We defined ‘early mortality’ as mortality within seven days of CRRT initiation. In addition, we defined ‘very early mortality’ as mortality within 24 h of CRRT initiation. This study was approved by the Institutional Review Board of Ewha Womans University, College of Medicine, and informed consent was waived because it was a retrospective cohort study.

### 2.2. Data Collection

Baseline characteristics were age, sex, body mass index (BMI), systolic blood pressure (SBP), diastolic blood pressure (DBP), mean arterial pressure (MAP), heart rate, comorbidities, Charlson Comorbidity Index (CCI) [23], SOFA score, and laboratory diagnostic data collected at the start of CRRT. Moreover, estimated glomerular filtration rate (eGFR) was calculated using the IDMS-traceable Modification of Diet in Renal Disease equation [24]. The presence of systemic inflammatory response syndrome (SIRS) [25]. APACHE II score and one-hour urine output immediately before CRRT initiation was also investigated. As a parameter for acute lung injury (ALI), patients with PaO_2_/FiO_2_ ≤ 300 mmHg were evaluated. Data from patients collected until 28 days after CRRT initiation was used, and their survival or all-cause mortality was examined during this period.

### 2.3. CRRT Protocol

The decision to initiate the CRRT and the CRRT settings of target clearance, blood flow, dialysate, and replacement fluid rates, and anticoagulation administration were determined through discussion and consultation with nephrologists. The criteria for CRRT initiation were medically intractable or persistent electrolyte imbalance and/or metabolic acidosis, and decreased urine output with volume overload and/or progressive azotemia. Hemodynamic instability was also an important indication. Generally, vascular access for CRRT was via a femoral venous catheter, and the predilution method of continuous venovenous hemodiafiltration was usually performed. Blood flow was gradually increased from an initial rate of 100 to 150 mL/min according to the hemodynamic status of the patient. Although the target clearance was 35–40 mL/kg/h in most patients, this target was increased to 60 mL/kg/h or higher in patients with severe sepsis or septic shock if possible [26]. Additionally, the anticoagulant administered was selected by nephrologists, and they were dependent on bleeding tendency or contraindications to conventional heparin. After CRRT initiation, attending physicians and experienced nurses monitored the body weight, urine output, laboratory results, actual delivered dose, and the hemodynamic status of the patients, and discussed the results with nephrologists to maintain the adequacy of CRRT.

### 2.4. Statistical Analysis

Continuous variables are expressed as the mean and standard deviation (SD), and categorical variables as number and percentage. Chi-square tests for categorical variables and Student’s t-test for continuous variables were used to compare baseline data between the two groups. We also performed univariate and multivariate logistic regression analyses to determine the factors associated with early or very early mortality. All statistical analyses were performed using SPSS version 23 software (SPSS, Chicago, IL, USA), and all *p*-values were two-tailed, with a predetermined alpha level <0.05 being considered statistically significant.

## 3. Results

### 3.1. Baseline Characteristics

Baseline demographic and clinical characteristics of these study patients are described in Table 1. For the 240 patients assessed, the mean age was 65.8 ± 14.7 years, and 150 patients (62.5%) were male. Mean SBP, DBP, and MAP were 112.5, 64.1, and 80.2 mmHg, respectively. In addition, there were 45 patients (18.8%) who had MAPs of less than 65 mmHg. Of the patients, 128 (53.3%) had hypertension, 89 (37.1%) were diagnosed with diabetes mellitus (DM), and the mean CCI was 6.6 ± 2.3. The mean volume of 1-hour urine outputs before CRRT was 27.2 ± 56.8 mL, and the mean APACHE II and SOFA scores were 26.1 ± 6.8 and 11.6 ± 3.9, respectively.

When we divided these patients into two groups (early mortality vs. 7-day survival past CRRT initiation), 138 (54.2%) died within seven days following the start of CRRT, and 110 (45.8%) survived more than seven days following CRRT initiation. There were no significant differences in age, sex distribution, BMI, the prevalence of underlying diseases, CCI, or 1-h urine volume output at baseline between the two groups. The proportion of patients diagnosed with sepsis was also not significantly different between the two groups. 

However, SBP, DBP, and MAP were significantly lower in the patients that exhibited early mortality following CRRT initiation compared to 7-day survivors. Additionally, heart rate and APACHE II and SOFA scores were significantly higher in the early mortality group compared to those of 7-day survivors. Finally, we observed that a higher proportion of patients suffered from SIRS in the early mortality group compared to the 7-day survivor group. 

Table 2 shows laboratory data of the patients at baseline. The mean white blood cell count was 13,100/µL; the mean hemoglobin was 9.4 ± 2.1 g/dL; and serum sodium, potassium, and bilirubin levels were 138.9 ± 7.3, 4.5 ± 1.0, and 2.8 ± 4.9 mEq/L, respectively. Additionally, the mean aspartate transaminase (AST) and alanine transaminase (ALT) levels were 401.8 ± 1157.2 and 158.8 ± 575.5 IU/L, respectively, and the mean eGFR was 22.7 ± 17.1 ml/min/1.73 m^2^. The mean arterial pH was 7.29 ± 0.13, and the base excess was −7.74 ± 7.10 mmol/L. When these data were compared between the early mortality and the 7-day survivor groups, serum phosphate level was significantly higher, while arterial pH was significantly lower in the early mortality group compared to the survivor group. Moreover, there was a higher proportion of patients with pH < 7.35 in the early mortality group than in the survivor group, and base excess was lower (base excess = −8.88) in the early mortality group compared to the survivor group (base excess = −6.43). Meanwhile, there was no difference in the proportion of patients with PaO_2_/FiO_2_ ≤ 300 mmHg between groups. The baseline characteristics and laboratory findings of patients with very early mortality were additionally described in Supplementary Materials (Tables S1 and S2).

### 3.2. Factors Associated with Early Mortality 

**Figure 1:**
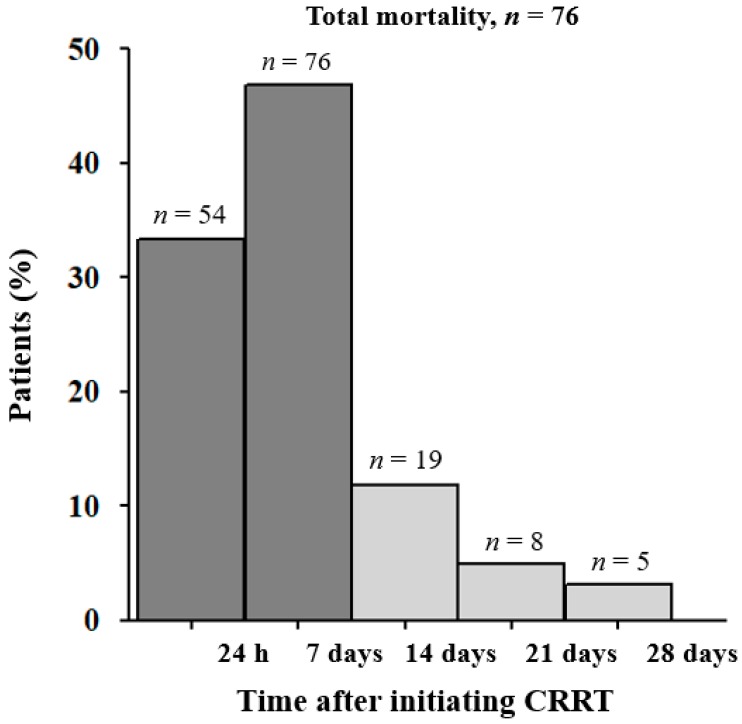
Mortality rate according to time after continuous renal replacement therapy (CRRT) initiation.

In this study, 162 patients (67.5%) died in the 28 days following CRRT initiation, and most of those deaths occurred in the early period following CRRT initiation (Figure 1). Specifically, 130 patients (80.2%) died within seven days following CRRT initiation, with 54 of those patients dying within 24 h following CRRT initiation. 

By univariate logistic regression analysis, patients with MAP < 65 mmHg and SIRS had an odds ratio (OR) of 2.436 (95% CI; 1.206–4.922, *p =* 0.013) and OR of 2.096 (95% CI; 1.054–4.168, *p =* 0.035) for an increased risk of early mortality, compared to patients with MAP > 65 mmHg, and an absence of SIRS. Moreover, a 1-SD increase of serum phosphate was significantly associated with an increased risk of early mortality (OR = 1.393, 95% CI (1.044–1.858), *p =* 0.024), and a 1-SD increase in SOFA score was also significantly associated with an increased incidence of early mortality (OR = 1.992, 95% CI (1.488–2.668), *p* < 0.001). Finally, the patients with pH < 7.35 also had a higher risk of early mortality compared to those with pH > 7.35 (OR = 4.326, 95% CI (2.409–7.768), *p* < 0.001). Importantly, after adjustment for demographic factors and other factors found to associate by univariate analysis with early mortality, increased SOFA score, low MAP (<65 mmHg), and low arterial pH (<7.35) all remained significantly associated with an increased risk of early mortality (SOFA score; OR = 1.758, 95% CI (1.282–2.412), *p* < 0.001, low MAP; OR = 2.771, 95% CI (1.213–6.327), *p =* 0.016, and low pH; OR = 3.067, 95% CI (1.593–5.903), *p =* 0.001) (Table 3). With consideration for overlapping parts between MAP, SIRS, and SOFA scores, additional multivariate regression analyses were performed by adjusting one of these three parameters to add to the other variables. As a result, SIRS showed a loss of significance, even in the analysis, without adjustment with MAP and SOFA scores.

We also performed multivariate logistic regression analyses to assess factors associated with very early mortality, to determine the similarities and differences between those factors related to the increased early mortality. We found that an increased serum sodium level, phosphate level, SOFA score, and low MAP (<65 mmHg) were all significantly associated with increased very early mortality, even after multivariate adjustment, as described previously (Table 4). Therefore, low MAP and increased SOFA scores are associated with an increased risk of both early and very early mortality. 

## 4. Discussion

This study demonstrated that early mortality within seven days following CRRT initiation was high in critically ill patients undergoing CRRT (54.2%). Moreover, MAP < 65 mmHg, arterial pH < 7.35, and high SOFA score at CRRT initiation significantly associated with increased risk of early mortality in these patients.

When we stratified the mortality rate of critically ill patients undergoing CRRT initiation, 80.2% of the total 162 patients that died during the 28-day follow-up period died within seven days following CRRT initiation, and 33.3% (54/162 patients) died within 24 h following CRRT initiation. A smaller percentage of patients, 19.8% (32/162 patients), died between eight and 28 days following CRRT initiation. Thus, we assessed which factors were associated with early or very early mortality in this group.

There are many studies assessing prognostic factors for the mortality risk of CRRT to predict and prevent poor clinical outcomes [13,14,15,16,17,18,19,20,21,22]. However, most of these studies assessed mortality beyond 28 days post-initiation of CRRT. Only a few studies have been conducted to investigate early mortality among critically ill patients undergoing CRRT [27,28]. In contrast, clinicians are often challenged to determine the benefit of CRRT, and it is difficult to identify patients that are more likely to demonstrate poor clinical outcomes, despite active treatment with CRRT to make the best decision for patients requiring RRT.

In this study, MAP < 65 mmHg, arterial pH < 7.35, and high SOFA score at CRRT initiation were risk factors for early mortality. Additionally, MAP < 65 mmHg, increased serum sodium, and phosphate levels, and high SOFA score at CRRT initiation, were significantly associated with an increased rate of very early mortality. Above these factors can be found in the other studies which were performed for the 28-, 60-, or 90-day mortality, which means that such factors may be more likely to be issued for early mortality.

CRRT treatment is primarily considered for patients in critical condition, who are hemodynamically unstable, and/or who suffer from increased intracranial pressure due to acute brain injury [29,30]; thus, the mortality rate of patients requiring CRRT is generally high. The mortality rate within hours or days following CRRT initiation is particularly high, mainly due to the poor condition of the patients at the initiation of CRRT. Specifically, Passos et al. [31] demonstrated a 7-day mortality of 45.0% (84/186 patients), and Prasad et al. [27] reported that 16.0% (17/106 patients) died within 24 h after the start of CRRT. In this study, 22.5% (54/240 patients) died within 24 h, and 54.2% (130/240 patients) died within seven days following CRRT initiation. Moreover, 80.2% of the total number of patients that died within the 28-day period (130/162 patients) died within 7 days following CRRT initiation. To our knowledge, this is the first study comparing the mortality rate at different time periods following CRRT initiation, and these results suggest that a majority of patients undergoing CRRT die within seven days following initiation. However, a prospective cohort study with a larger population should be performed to confirm these results.

AKI, combined with cardiovascular instability, fluid overload, cerebral edema, and high fluid requirement, generally indicates a need for CRRT [32,33]. In addition, the need to eliminate inflammatory mediators, remove fluid, or eliminate other endogenous toxic solutes have been presented as non-renal reasons to initiate CRRT [34]. In our study, several factors were found to be associated with early or very early mortality in patients undergoing CRRT. MAP is a hemodynamic parameter, and maintaining MAP ≥ 65 mmHg is recommended in the management of patients with septic shock, a condition for which CRRT is a common treatment [35]. In addition, a SOFA score represents a severity parameter and it is a widely accepted prognostic factor for critically ill patients [36,37]. Several studies report a significant association between a high SOFA score at CRRT initiation, and increased mortality [38,39,40]. Arterial pH is one of the variables that is considered in the APACHE II score, which was designed to measure the disease severity and the risk of death in critically ill patients, and several studies have demonstrated that arterial pH is associated with increased mortality in patients undergoing CRRT [41,42,43]. In addition, previous studies have reported that hypernatremia and hyperphosphatemia are common in critically ill patients, and they were associated with increased morbidity and mortality [44,45,46,47]. The factors identified in this study could be predictable through previous studies; however, this study has significance because it reaffirms the clinical importance of these factors by assessing the association with early death showing high mortality rate. The results presented here do not indicate the futility of CRRT treatment for patients with lower MAP, lower pH, higher serum sodium or phosphate, or high SOFA score. However, these results could be useful in predicting the prognosis of critically ill patients after CRRT initiation.

There are some limitations to our study. First, this was a single center study with a relatively small sample size, so we cannot rule out selection bias, and these results may not be generalizable to other populations. Therefore, a future multiple-center study with a larger sample size is warranted to verify factors that are associated with early mortality in critically ill patients undergoing CRRT. Second, because of the inherent limitations of a retrospective study, other potential factors associated with early death in critically ill patients, such as causes of CRRT initiation or primary diagnosis at admission may not have been assessed. Third, we investigated mortality events based on arbitrarily stratified time periods, such as within 24 h, 7 days, or 28 days following CRRT initiation, and defined ‘early mortality’ or ‘very early mortality’ discretionally. However, several studies for mortality of the patients undergoing CRRT have used 28-days mortality as the end-point and there have been also some studies for 24-hour and 7-day mortality, so that these timeframes are not without precedent. Moreover, in this study, we found that the highest mortality following CRRT occurred in the early timeframe following CRRT initiation. Lastly, this observational study does not allow us to conclude a causal relationship, and it only demonstrates the associations between clinical factors and early or very early mortality in patients undergoing CRRT treatment.

## 5. Conclusions

In conclusion, we found that the early mortality rate within seven days following CRRT initiation was very high in this cohort of critically ill patients undergoing CRRT. Moreover, low MAP, low arterial pH, and high SOFA score at CRRT initiation were associated with early mortality in these patients. Although these factors may not be used as determinants in deciding whether or not CRRT should be initiated in critically ill patients, they may be useful in predicting early or very early mortality despite active treatment with CRRT.

## Figures and Tables

**Table 1:** Baseline characteristics of study patients.

Variables	Total	7-Day Mortality	7-Day Survivors	*p* Value
	(*n* = 240, 100%)	(*n* = 130, 54.2%)	(*n* = 110, 45.8%)	
Age (year)	65.8 ± 14.7	65.9 ± 14.2	65.7 ± 15.3	0.928
Male sex, *n* (%)	150 (62.5)	78 (60.0)	72 (65.5)	0.231
BMI (kg/m^2^)	23.1 ± 4.3	23.0 ± 4.7	23.1 ± 3.7	0.850
SBP (mmHg)	112.5 ± 23.9	107.3 ± 22.6	118.6 ± 24.2	<0.001
DBP (mmHg)	64.1 ± 15.2	61.9 ± 14.1	66.8 ± 16.1	0.013
MAP (mmHg)	80.2 ± 16.0	77.0 ± 15.1	84.0 ± 16.3	0.001
MAP < 65 mmHg, *n* (%)	45 (18.8)	32 (24.6)	13 (11.8)	0.008
Heart rate (per min)	107.2 ± 24.0	110.7 ± 22.5	103.1 ± 25.2	0.015
Comorbidity disease				
Hypertension, *n* (%)	128 (53.3)	65 (50.0)	47 (42.7)	0.160
Diabetes mellitus, *n* (%)	89 (37.1)	43 (33.1)	46 (41.8)	0.103
CHF, *n* (%)	15 (6.3)	9 (6.9)	6 (5.5)	0.423
COPD, *n* (%)	4 (1.7)	2 (1.8)	2 (1.5)	0.624
Age CCI	6.60 ± 2.31	6.50 ± 2.42	6.72 ± 2.18	0.468
SIRS, *n* (%)	199 (82.9)	114 (87.7)	85 (77.3)	0.025
Sepsis, *n* (%)	75 (31.3)	42 (32.3)	33 (30.0)	0.404
Amount of 1-h UO (mL)	27.2 ± 56.8	26.6 ± 60.1	27.8 ± 52.9	0.879
APACHE II score	26.1 ± 6.8	28.9 ± 6.2	22.9 ± 6.0	<0.001
SOFA score	11.58 ± 3.87	12.70 ± 3.53	10.27 ± 3.86	<0.001

Data are presented as mean ± standard deviation or number (%). Abbreviations: BMI, body mass index; SBP, systolic blood pressure; DBP, diastolic blood pressure; MAP, mean arterial blood pressure; CHF, congestive heart failure; COPD, chronic obstructive heart failure; CCI, Charlson comorbidity index; SIRS, systemic inflammatory response syndrome; UO, urine output.

**Table 2:** Baseline laboratory data of study patients.

Variables	Total	7-Day Mortality	7-Day Survivors	*p* Value
	(*n* = 240, 100%)	(*n* = 130, 54.2%)	(*n* = 110, 45.8%)	
WBC (103/µL)	13.1 ± 12.3	14.1 ± 15.5	11.9 ± 7.1	0.147
Hemoglobin (g/dL)	9.4 ± 2.1	9.5 ± 2.3	9.4 ± 1.9	0.892
Platelet (103/µL)	127.8 ± 86.5	127.9 ± 89.1	127.6 ± 83.8	0.980
Sodium (mEq/L)	138.9 ± 7.3	139.6 ± 7.9	138.1 ± 6.4	0.119
Potassium (mEq/L)	4.5 ± 1.0	4.6 ± 1.0	4.5 ± 0.9	0.289
Calcium (mg/dL)	7.8 ± 1.3	7.7 ± 1.2	7.8 ± 1.4	0.669
Phosphate (mg/dL)	5.5 ± 2.9	5.9 ± 3.2	5.0 ± 2.5	0.021
Bilirubin, total (mg/dL)	2.8 ± 4.9	2.9 ± 4.9	2.6 ± 4.8	0.568
AST (IU/L)	401.8 ± 1157.2	416.8 ± 1012.7	384.5 ± 1308.8	0.831
ALT (IU/L)	185.8 ± 575.5	199.1 ± 473.4	170.6 ± 675.7	0.706
eGFR (ml/min/1.73 m^2^)	22.7 ± 17.1	23.7 ± 16.2	21.4 ± 18.1	0.300
pH	7.29 ± 0.13	7.25 ± 0.12	7.33 ± 0.12	<0.001
pH < 0.35, *n* (%)	164 (68.3)	107 (82.3)	57 (51.8)	<0.001
BE (mmol/L)	−7.75 ± 7.10	−8.88 ± 6.64	−6.43 ± 7.43	0.008
PaO_2_/FiO_2_ < 300	188 (78.3)	106 (81.5)	82 (74.5)	0.125

Data are presented as mean ± standard deviation or number (%). Abbreviations: WBC, whole blood cell; AST, aspartate aminotransferase; ALT, alanine aminotransferase; eGFR, estimated glomerular filtration rate; BE, base excess.

**Table 3:** Logistic regression analysis for early mortality *.

	Univariate		Multivariate	
Factors	OR (95% CI)	*p* Value	OR (95% CI)	*p* Value
**Age (per 1-SD increase)**	1.012 (0.785−1.305)	0.928	1.079 (0.803−1.450)	0.614
**Male (versus Female)**	0.792 (0.467−1.341)	0.385	0.877 (0.477−1.610)	0.671
**BMI (per 1-SD increase)**	0.976 (0.757−1.259)	0.976	0.929 (0.695−1.240)	0.616
**MAP < 65 mmHg (versus MAP ≥ 65 mmHg)**	2.436 (1.206−4.922)	0.013	2.771 (1.213−6.327)	0.016
**SIRS (versus no SIRS)**	2.096 (1.054−4.168)	0.035	1.602 (0.717−3.583)	0.251
**Sepsis (versus no sepsis)**	0.898 (0.519−1.555)	0.898	-	-
**Phosphate (per 1-SD increase)**	1.393 (1.044−1.858)	0.024	1.197 (0.869−1.649)	0.270
**pH < 7.35 (versus pH ≥ 7.35)**	4.326 (2.409−7.768)	<0.001	3.067 (1.593−5.903)	0.001
**SOFA score (per 1-SD increase)**	1.992 (1.488−2.668)	<0.001	1.758 (1.282−2.412)	<0.001

* The early mortality was defined by the death within seven days after CRRT initiation. Abbreviations: OR, odds ratio; CI, confidential interval; SD, standard deviation; BMI, body mass index; MAP, mean arterial blood pressure; SIRS, systemic inflammatory response syndrome

**Table 4:** Logistic regression analysis for very early mortality *.

	Univariate		Multivariate	
Factors	OR (95% CI)	*p* Value	OR (95% CI)	*p* Value
**Age (per 1-SD increase)**	1.069 (0.785−1.456)	0.672	1.363 (0.909−2.043)	0.134
**Male (versus female)**	0.632 (0.337−1.151)	0.131	0.707 (0.327−1.532)	0.380
**BMI (per 1-SD increase)**	1.038 (0.768−1.402)	0.810	0.956 (0.657−1.391)	0.816
**Diabetes mellitus (versus non-diabetes)**	0.354 (0.172−0.730)	0.005	0.456 (0.189−1.101)	0.081
**MAP < 65 mmHg (versus MAP ≥ 65 mmHg)**	4.295 (2.144−8.607)	<0.001	8.498 (3.379−21.375)	<0.001
**SIRS (versus no SIRS)**	2.352 (0.874−6.326)	0.090	-	-
**Sepsis (versus no sepsis)**	1.103 (0.570−2.136)	0.770	-	-
**Sodium (per 1-SD increase)**	1.458 (1.202−1.769)	<0.001	1.588 (1.241−2.031)	<0.001
**Phosphate (per 1-SD increase)**	1.632 (1.207−2.207)	0.001	1.669 (1.149−2.424)	0.007
**pH < 7.35 (versus pH ≥ 7.35)**	4.828 (1.965−11.863)	0.001	2.395 (0.860−6.675)	0.095
**SOFA score (per 1-SD increase)**	1.895 (1.329−2.702)	<0.001	1.691 (1.049−2.725)	0.031

* Very early mortality was defined by death within 24 h after CRRT initiation. Abbreviations: OR, odds ratio; CI, confidential interval; SD, standard deviation; BMI, body mass index; MAP, mean arterial blood pressure; SIRS, systemic inflammatory response syndrome

## Supplementary Materials

The following are available online at http://www.mdpi.com/2077-0383/7/10/334/s1, Table S1: Baseline characteristics of study subjects, Table S2: Laboratory data of study subjects at baseline.
