# Supplementary material for: Factors Associated with Early Mortality in Critically Ill Patients Following the Initiation of Continuous Renal Replacement Therapy
Source: J Clin Med. 2018 Oct 8;7(10):334. doi: 10.3390/jcm7100334 (PMC6210947; doi:10.3390/jcm7100334)
Supplement: Supplementary file 1 [file jcm-07-00334-s001.pdf]

Table S1. Baseline characteristics of study subjects.

Variables	Total (n = 240)	Mortality within 24 hours (n = 54)	Survivors for 24 hours (n = 186)	*P* value
Age (year)	65.8 ± 14.7	66.5 ± 14.9	65.6 ± 14.6	0.673
Male sex, n (%)	150 (62.5)	29 (53.7)	121 (65.1)	0.088
BMI (kg/m^2)	23.1 ± 4.3	23.2 ± 4.8	23.0 ± 4.1	0.811
SBP (mmHg)	112.5 ± 23.9	97.9 ± 18.2	116.7 ± 23.8	<0.001
DBP (mmHg)	64.1 ± 15.2	57.9 ± 11.4	65.9 ± 15.7	<0.001
MAP (mmHg)	80.2 ± 16.0	71.2 ± 11.6	82.9 ± 16.2	<0.001
MAP<65mmHg, n (%)	45 (18.8)	21 (38.9)	24 (12.9)	<0.001
Heart rate (per min)	107.2 ± 24.0	112.2 ± 23.1	105.7 ± 24.1	0.079
Comorbidity disease				
Hypertension, n (%)	128 (53.3)	28 (51.9)	100 (53.8)	0.462
Diabetes mellitus, n (%)	89 (37.1)	11 (20.4)	78 (41.9)	0.003
CHF, n (%)	15 (6.3)	3 (5.6)	12 (6.5)	0.553
COPD, n (%)	4 (1.7)	0 (0.0)	4 (2.2)	0.358
Age CCI	6.6 ± 2.3	6.35 ± 2.25	6.67 ± 2.33	0.372
SIRS, n (%)	199 (82.9)	49 (90.7)	150 (80.6)	0.058
Amount of 1-h UO (mL)	27.2 ± 56.8	29.0 ± 79.2	26.6 ± 48.9	0.791
APACHE II score	26.1 ± 6.8	29.9 ± 6.0	25.0 ± 6.6	<0.001
SOFA score	11.6 ± 3.9	13.3 ± 3.1	11.1 ± 3.9	<0.001

Data are presented as mean ± standard deviation or number (%). Abbreviations: BMI, body mass index; SBP, systolic blood pressure; DBP, diastolic blood pressure; MAP, mean arterial blood pressure; CHF, congestive heart failure; COPD, chronic obstructive heart failure; CCI, Charlson comorbidity index; SIRS, systemic inflammatory response syndrome; UO, urine output

Table S2. Laboratory data of study subjects at baseline.

Variables	Total	Mortality within 24hours	Survivors for 24 hours	*P* value
	(n = 240)	(n = 54)	(n = 186)	
WBC (10^3/μL)	13.1 ± 12.3	15.9 ± 2.2	11.1 ± 0.8	0.558
Hemoglobin (g/dL)	9.4 ± 2.1	9.4 ± 2.5	9.5 ± 2.0	0.775
Platelet (10^3/μL)	127.8 ± 86.5	120.5 ± 86.9	129.9 ± 86.5	0.482
Sodium (mEq/L)	138.9 ± 7.3	142.4 ± 8.6	137.9 ± 6.5	0.001
Potassium (mEq/L)	4.5 ± 1.0	4.7 ± 1.1	4.5 ± 0.9	0.169
Calcium (mg/dL)	7.8 ± 1.3	7.6 ± 1.0	7.8 ± 1.4	0.485
Phosphate (mg/dL)	5.5 ± 2.9	6.7 ± 3.8	5.1 ± 2.5	0.007
Bilirubin, total (mg/dL)	2.8 ± 4.9	3.4 ± 6.5	2.6 ± 4.4	0.319
AST (IU/L)	401.8 ±1157.2	544.4 ± 1096.9	360.8 ± 1173.6	0.310
ALT (IU/L)	158.8 ± 575.5	292.7 ± 619.1	154.9 ± 560.2	0.149
eGFR (ml/min/1.73 m^2)	22.7 ± 17.1	25.2 ± 15.9	21.9 ± 17.4	0.215
pH	7.29 ± 0.13	7.22 ± 0.14	7.30 ± 0.12	<0.001
pH<0.35, n (%)	164 (68.3)	48 (88.9)	116 (62.4)	0.001
BE (mmol/L)	-7.74 ± 7.10	-10.0 ± 7.5	-7.1 ± 6.9	0.008
PaO_2/FiO_2 <300 mmHg	188 (78.3)	43 (49.6)	145 (78.0)	0.478

Data are presented as mean ± standard deviation or number (%). Abbreviations: WBC, whole blood cell; AST, aspartate aminotransferase; ALT, alanine aminotransferase; eGFR, estimated glomerular filtration rate; BE, base excess.
